# Simulation of the Ontogeny of Social Jet Lag: A Shift in Just One of the Parameters of a Model of Sleep-Wake Regulating Process Accounts for the Delay of Sleep Phase Across Adolescence

**DOI:** 10.3389/fphys.2018.01529

**Published:** 2018-11-05

**Authors:** Arcady A. Putilov, Evgeniy G. Verevkin

**Affiliations:** ^1^Research Group for Math-Modeling of Biomedical Systems, Research Institute for Molecular Biology and Biophysics, Novosibirsk, Russia; ^2^Laboratory of Sleep/Wake Neurobiology, Institute of Higher Nervous Activity and Neurophysiology of the Russian Academy of Sciences, Moscow, Russia

**Keywords:** somnostat, two-process model, sleep debt, simulation, sleep-wake regulation, maturational changes

## Abstract

The term “social jet lag” was introduced for defining the conflict between social and biological clocks due to the general practice of shifting weekday risetime on early morning hours. The phase delay of the sleep-wake cycle during adolescence is one of the most remarkable features of the ontogenesis of sleep that is incompatible with early school start times. It was previously proposed that the process of accumulation of sleep pressure during wakefulness is slowing down in post-pubertal teens to allow them to stay awake for a longer period of time thus causing the delay of their bedtime. In order to examine this proposition, we traced the ontogeny of social jet lag using sleep times reported for 160 samples of study participants of different ages as an input to a model of sleep-wake regulatory process. The simulations suggested that a gradual change in just one of the model’s parameters, the time constant of wakefulness phase of the sleep-wake regulatory process, might explain the association of the transition between childhood and adulthood with the prolongation of time staying awake, delay of sleep time, and reduction of sleep duration. We concluded that the implication of the sleep-wake regulating model would be of help for understanding precisely how social jet lag varies with age and what are the chronophysiological causes of this variation.

## Introduction

Epidemic of sleep deprivation among adolescents when schedules maintained during the school year are resulted in insufficient and ill-timed sleep was recognized in many post-industrial societies with different cultural traditions (Spruyt et al., 2005; Chung and Cheung, 2008; Liu et al., 2008; Carskadon, 2011; Kim et al., 2011; Perkinson-Gloor et al., 2013; Short et al., 2013; Borisenkov et al., 2016; Carissimi et al., 2016; Komada et al., 2016; Gariépy et al., 2017). The explanations of this phenomenon relay on pubertal changes in the homeostatic and circadian regulation of sleep (Andrade et al., 1993; Thorleifsdottir et al., 2002; Gau and Soong, 2003; Carskadon et al., 2004; Jenni and O’Connor, 2005; Millman, 2005; Hagenauer et al., 2009; Urner et al., 2009; Gradisar et al., 2011). The most remarkable characteristic of such changes is a delayed sleep phase that is incompatible with early school start times (see, e.g., Hagenauer et al., 2009, for review).

Wittmann et al. (2006) introduced the term “social jet lag” to conceptualize the conflict between social and biological clocks and to propose a methodology for assessment of its severity (originally by means of a questionnaire). The results of such assessments supported the concern about exaggeration of social jet lag due to a confrontation between early school times and adolescents’ biological tendency to delay (see, e.g., Crowley et al., 2014). From a school educational perspective a high susceptibility of adolescents to social jet lag may seem disappointing as being at school age means inability to perform optimally at school in the morning. It is not clearly understood why adolescents are often more susceptible to social jet lag than either young adults or children, even after accounting for differences between ages in sleep duration.

Many researchers reported the estimates of sleep times on weekday and weekend for study participants of different ages. These reports provided an empirical basis for our present attempt to trace the ontogeny of social jet by applying our quantitative model of sleep-wake regulatory process. Here, we proposed a methodological framework for quantitative prediction of social jet lag during ontogeny. We argued that the implication of this model of sleep-wake regulation would provide understanding of how social jet lag varies with age and what are the chronophysiological underpinnings of this variation.

In particular, our simulations were aimed on clarification of a question of which of two basic mechanisms of sleep-wake regulation – homeostatic or circadian – is the major contributor to sleep phase delay in late adolescence. The model-based simulation can help tin explaining why it need not propose the phase delay of circadian pacemaker for prediction of the amount of social jet lag at this age. In mammals, the “master” clock is seated in the suprachiasmatic nuclei of the hypothalamus and its major function is to entrain the circadian period and to reset the circadian phase of all other structures and processes of an organism in response to changes in the environmental 24-h light–dark cycle (Moore and Eichler, 1972; Ibuka and Kawamura, 1975; Ralph et al., 1990). Earlier research did not question the possibility that the developmental changes in the period and phase of this clock mechanism underlie the dramatic changes in sleep phase across adolescence. Some of previously published reports provided experimental support for this possibility (e.g., Carskadon et al., 2004). For example, Carskadon et al. (1999) showed that adolescents have a free-running circadian period that is significantly longer than that found in adults using similar protocols (24.27 vs. 24.12 h). However, evidence accumulating by more recent research allows the conclusion that a phase delay of circadian pacemaker cannot account for the delaying shift in sleep times across adolescence. For instance, the most recent report by Crowley and Eastman (2018) did not provide evidence for any difference between free-running circadian periods of adolescents aged 14–17 years and adults aged 30–45 years. Moreover, earlier cross-sectional (e.g., Czeisler et al., 1999) and longitudinal studies (e.g., Kendall et al., 2001) of adults did not reveal age-associated change in the free-running circadian period.

Therefore, empirical evidence indicates that the mechanism of homeostatic rather than circadian regulation of sleep might underlies the observed delay of sleep phase in adolescents. In the classical two-process model of human sleep regulation, this mechanism is viewed as a two-phase (normally 24-h) cycle of alternations in sleep pressure with the phase of its reverse-exponential buildup during wake episode and the following phase of its exponential decay throughout a sequence of non-rapid eye movement (NREM) sleep episodes (Daan et al., 1984). The results of longitudinal study reported by Crowley et al. (2014) were interpreted as evidencing for more slow buildup of sleep pressure in older than younger adolescents. Consequently, sleep pressure reaches its everyday maximum later leading to the delayed bedtime in older adolescents. By contrast, such a marker of circadian phase as evening onset of melatonin secretion remained relatively stable across the study (Crowley et al., 2014). These findings were in agreements with the previous reports indicating changes in accumulation of sleep pressure across waking, on the one hand, and the absence of changes in the decay of homeostatic sleep pressure during sleep, on the other hand (Jenni et al., 2005; Campbell et al., 2011; Tarokh et al., 2012). For instance, in the study reported by Jenni et al. (2005) the decay of sleep pressure was indexed by the rate of decline of slow-wave activity (SWA) across NREM sleep episodes shown before and after 36 h of sleep deprivation. The simulation of these data suggested an increase in the time constant of the buildup phase in post-pubertal versus pre-pubertal teens while the time constant for the decay phase remained unchanged across these adolescent’s ages.

Consequently, in our simulations we accounted for these empirical observations. Namely, we did not expect that changes in sleep times during the transition from childhood to adulthood might be linked to any changes in parameters of the circadian regulation of the sleep-wake cycle. Instead, we tested the hypothesis proposing sole a change in, might be, just one of the parameters of homeostatic regulation, such as the time constant of the reverse-exponential phase of accumulation of sleep pressure across wake episode that we expected to increase during ontogeny.

## Materials and Methods

**FIGURE 1:**
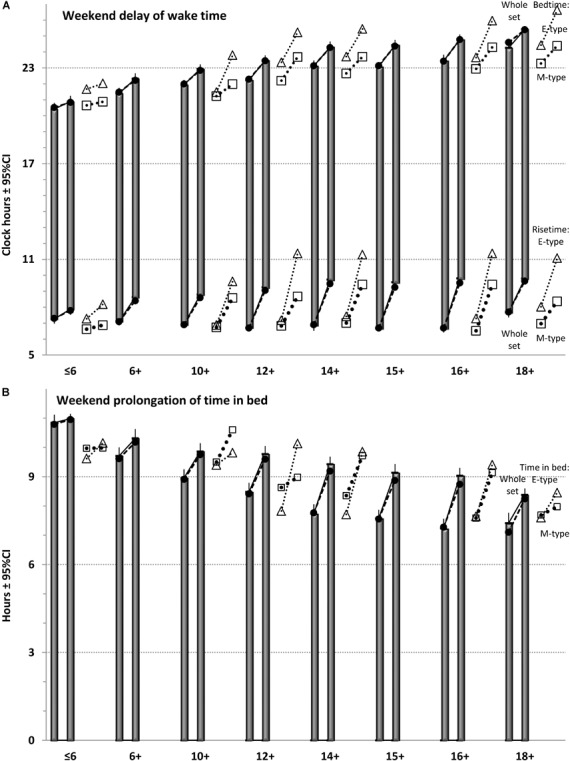
Sleep times in eight age groups. The samples of the whole set (*n* = 160) were sorted into eight age groups. Closed circles: simulated sleep times. Mean: averaged sleep times. M- and E-types: sleep times for two chronotypes (open triangles and squares, respectively) are shown on the right side from averaged sleep times. **(A)** Times of wakefulness. **(B)** Time in bed.

**FIGURE 2:**
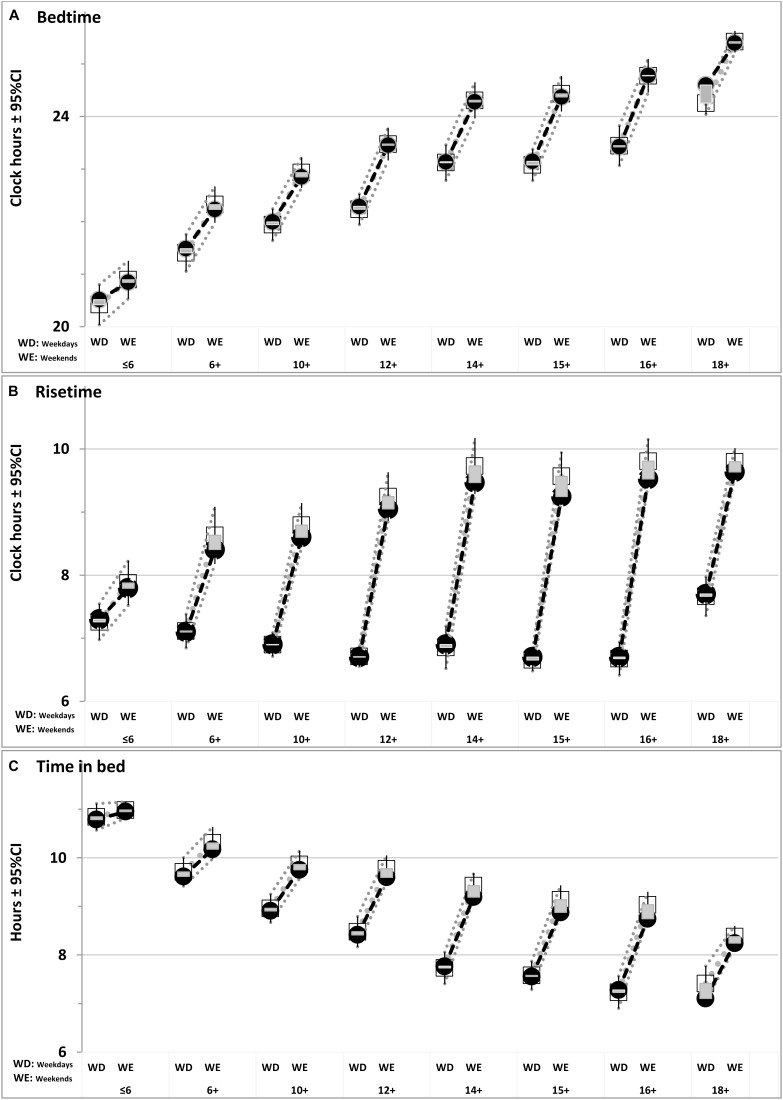
Comparison of simulated sleep times with ranges of their empirical variation. Empirical data are represented by age-averaged values (open squares) with 95% Confidence Interval (±95%CI). Shadowed areas denote their deviations from simulated values (closed circles) given in Table 3. **(A–C)** Bedtime, Risetime, and Time in bed.

We identified the reported estimates of sleep times in more than 60 previously published journal papers (see Supplementary Material). Mean ages for 160 samples of study participants varied between 0.5 and 23.4 years. The subdivision of the whole set of samples into sub-samples representing different ages gave eight subsets of sleep times shown in Figures 1, 2 and Tables 1, 2. School age participants predominated in the whole set of 160 collected samples (4/5) due to the concern of many sleep researchers about epidemic of sleep deprivation among adolescents. Therefore, non-equal intervals of ages were used for subdivision into these eight subsets. Sleep times for workdays/schooldays and free days were mostly retrospectively self-reported by study participants, while the estimates based on sleep diary and actigraphy were collected for a rather small fraction of samples (see Supplementary Material).

**Table 1:** List of 13 parameters of the rhythmostat model utilized for simulating sleep times.

Simulated data	Putilov	Wolfson et al.	Range
**Sine wave-form circadian modulation (2):**			
*A* (circadian amplitude), relative SWA	0.50	0.50	0.50–0.50
φ_0_ (initial circadian phase), radians	4.13	3.66	3.66–3.66
*τ* (entrained circadian period), hours	24.00	24.00	24.00–24.00
*k* (2-fold increase of the circadian term)	2.00	2.00	2.00–2.00
**Exponential buildup (1a) and decay (1b) phases:**			
SWA_l_ (lower asymptote), relative SWA	0.70	0.70	0.70–0.70
SWA_b_ (lowest decay), relative SWA	0.75	0.75	0.755–0.765
SWA_d_ (highest buildup), relative SWA	2.50	2.50	2.75–3.25
SWA_u_ (upper asymptote), relative SWA	4.50	4.50	5.00–6.00
*T_d_* (decay phase constant), hours	1.95	2.29	2.36–2.61
*T_b_* (buildup phase constant), hours	27.04	25.22	18.39–27.81
**Initial times for buildup and decay phases:**			
*t2* (bedtime on free days, e.g., vacation), clock hours	23.00	24.20	20.84–25.40
*t1* (risetime on free days, e.g., vacation), clock hours	7.00	8.85	7.79–9.64
Risetime on weekdays, clock hours		6.33	6.70–7.70

**FIGURE 3:**
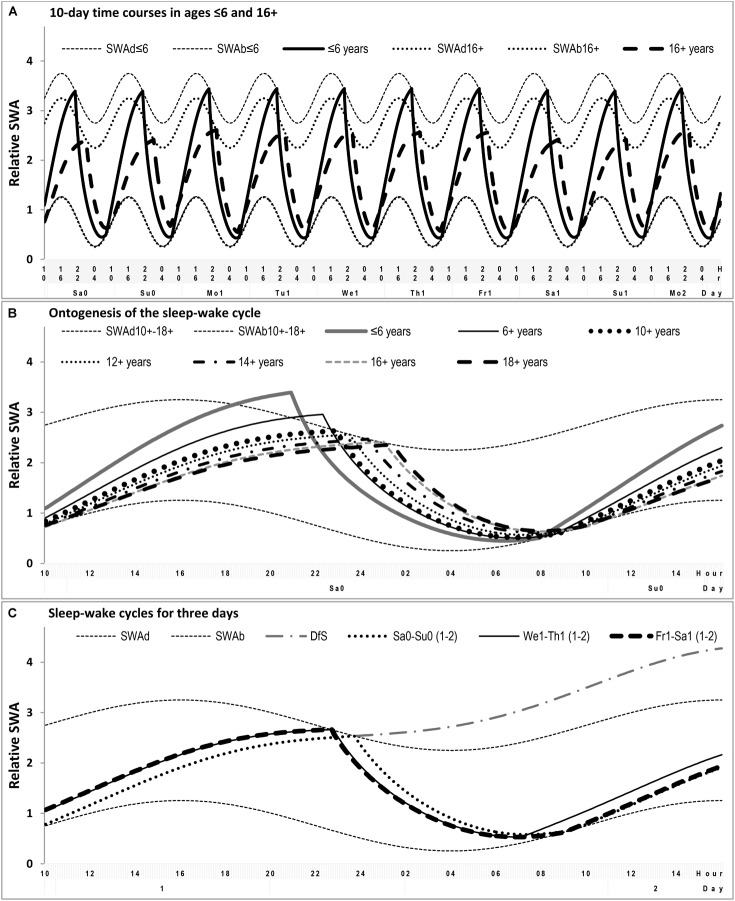
Simulation of the sequencies of sleep-wake cycles. **(A)** Ten consecutive sleep-wake cycles including two last free (e.g., vacation) days and the following workdays and wekends in simulations of two distant ages. **(B)** Comparison of the sleep-wake cycle on free days for seven different ages. **(C)** Comparison of the simulated sleep-wake cycles on free day, weekday and weekend days, the example of sleep times obtained by averaging over 160 samples. SWAd and SWAb: highest buildup and lowest decay of relative SWA, respectively; DfS: further buildup of SWA expected in the case of Deprivation from Sleep. Phases of the sleep-wake regulating process (1) simulated as alternations between exponential buldups and decays of SWA modulated by sine-form function with 24-h period (see the model’s parameters in (Tables 1, 2).

*Parameters of the model (1) applied for simulation of sleep times illustrated in Figures 1–3. Putilov: initial parameters derived in Putilov (1995) using data on sleep duration after extended wakefulness and on SWA in naps and extended sleep episodes (mean SWA = 1 in baseline night episode). Wolfson et al.: example of one of the studies in which three different methods of data collection (survey, diary, and actigraphy) were applied; Range: range of parameters’ values for eight subsets of the whole dataset representing eight age groups from age <6 years to age 18+ years*.

**Table 2:** Subset of nine parameters of the model varying across ages and averaged sleep times.

Age	≤6	6+	10+	12+	14+	15+	16+	18+
*n*	16	21	24	25	14	21	21	18
**Varying parameters used in simulations**					
SWA_b_ (lowest decay)	0.765	0.76	0.755	0.755	0.755	0.755	0.755	0.755
SWA_d_ (highest buildup)	3.25	3.00	2.75	2.75	2.75	2.75	2.75	2.75
SWA_u_ (upper asymptote)	6.00	5.50	5.00	5.00	5.00	5.00	5.00	5.00
*T_d_* (decay phase constant)	2.36	2.42	2.41	2.56	2.61	2.47	2.55	2.40
*T_b_* (buildup phase constant)	18.39	20.40	21.67	22.86	24.80	25.12	26.14	27.81
*t2* (bedtime on vacations)	20.84	22.23	22.85	23.45	24.28	24.37	24.78	25.40
*t1* (risetime on vacations)	7.79	8.41	8.60	9.05	9.47	9.25	9.53	9.64
Risetime on weekdays	7.30	7.10	6.90	6.70	6.90	6.70	6.70	7.70
**Betimes obtained by averaging within eight sub-samples**					
Bedtime on weekends	20.89	22.32	22.93	23.47	24.31	24.43	24.77	25.42
Risetime on weekends	7.88	8.63	8.79	9.25	9.73	9.57	9.81	9.80
Bedtime on weekdays	20.42	21.40	21.94	22.23	23.12	23.08	23.44	24.25
Risetime on weekdays	7.26	7.11	6.90	6.71	6.86	6.66	6.68	7.66

*In any of eight simulations, other five parameters remained the same as reported in Table 1. Moreover, changes in SWA levels were not necessary to simulate sleep times in most of age groups (ages from 10+ to 18+). See also Figures 1, 2 illustrating empirical and simulated sleep times for these eight age groups*.

For 13 of 160 samples (see Supplementary Material), sleep times were calculated by averaging over the times reported separately for M- and E- types classified with a morning-evening preference questionnaire (morning or early and evening or late chronotypes, respectively). Their sleep times are illustrated in Figure 1 to provide possibility to compare sleep times in these chronotypes with group-averaged sleep times obtained for the same and distant ages.

A sample from the report of Wolfson et al. (2003) was of special interest for the present simulations because sleep times of each of 302 high school students (grades 9–12) of their study were determined by three different methods, survey, diary, and actigraphy. Since sleep times for the vast majority of analyzed samples were obtained in survey, data of Wolfson et al. (2003) provided possibility to identify possible differences between subjective (survey) and objective (actigraphy) estimates with discrepancies between simulations and averaged empirical estimates. We averaged sleep times obtained with these different methods, simulated them, and calculated discrepancies between empirical and simulated values for each method of data collection (Table 3). In particular, we noted that Wolfson et al. (2003) obtained rather similar estimates for bedtime on weekends using the methods of survey and actigraphy. In contrast, a rather large difference was reported for risetime on weekends (Table 3). Compared to the actigraphy estimate, it seemed to be significantly overestimated (*p* < 0.001) in the survey.

**Table 3:** Discrepancies between empirical and simulated sleep times for different subsets.

Times		≤6	6+	10+	12+	14+	15+	16+	18+	Survey	Actigraphy
Bedtime	Weekdays	-0.12	-0.08	-0.05	-0.05	-0.01	-0.07	0.02	-0.34	-0.29	-0.07
	Weekends	0.05	0.09	0.08	0.02	0.03	0.06	-0.01	0.03	0.13	0.03
Risetime	Weekdays	-0.04	0.01	0.00	0.01	-0.04	-0.04	-0.02	-0.04	0.00	-0.03
	Weekends	0.08	0.23	0.19	0.20	0.26	0.32	0.28	0.16	0.85	-0.10
Time in bed	Weekdays	0.06	0.09	0.05	0.07	-0.03	0.02	-0.05	0.31	0.29	0.04
	Weekends	0.03	0.13	0.11	0.17	0.23	0.26	0.29	0.13	0.72	-0.13
Shift	Bedtime	-0.14	-0.17	-0.14	-0.08	-0.04	-0.13	0.03	-0.37	-0.41	-0.10
	Risetime	-0.12	-0.21	-0.19	-0.18	-0.31	-0.37	-0.30	-0.20	-0.84	0.07
	Time in bed	0.02	-0.04	-0.06	-0.11	-0.27	-0.24	-0.33	0.17	-0.43	0.17

*Discrepancies were calculated by subtracting simulated sleep times from empirical sleep times. See also the parameters for these simulations in Figures 1, 2 and Table 2 for comparison of these discrepancies with variation in empirical datasets. Survey and Actigraphy: Wolfson et al. (2003) obtained sleep times by using different methods of data collection; Time in bed: difference between rise-and bedtimes. Parameters of the model are given in Tables 1, 2. See also Figures 1, 2 for comparison of these discrepancies with variation in empirical datasets*.

The SPSS statistical software package (IBM, Armonk, NY, United States, version 22.0) was used for calculation of group-averaged values with 95% Confidence Interval (CI) illustrated (Figures 1, 2).

To simulate sleep times, we implemented our version of the two-process model of sleep-wake regulation (Putilov, 1995). The model and the way of derivation of its initial parameters (shown in Table 1, left column) are explained in more detail in Putilov (1995, 2014). The model was named “rhythmostat” (Putilov, 1995) because it describes in mathematic terms the homeostatic process (“somnostat” in Daan et al., 1984) which parameters are not constant but, rather, are modulated by the circadian pacemaker.

If *t1* and *t2* are the initial times for the buildup and decay phases (rise- and bedtime, respectively), this sleep-wake regulating process can be simulated using the following equations:

(1) $$X(t)=[X_{u}+C(t)]-\{[X_{u}+C(t)]-X_{b}\}*e^{-(t-t1)/[Tb-k*C(t)]}$$

(2) $$X(t)=[X_{l}+C(t)]-\{X_{d}[X_{l}+C(t)]\}*e^{-(t-t2)/[Tb-k*C(t)]}$$

where

(3) $$C(t)=A*\;\sin(2\text{π}*t/\tau +{\text{φ}}_{0})$$

is a periodic function with a period *τ* assigned to 24 h (Putilov, 1995).

This model was chosen because, the “classical” model of the homeostatic component of the sleep-wake regulating process (“somnostat”) does not account for the circadian modulation of this component. The weekday and weekend sleep episodes are started at different circadian clock times. Therefore, we expected that these two sleep episodes will be different in both intensity and duration due to the difference in modulating influence of the circadian pacemaker on the parameters of the exponential decay of the homeostatic component of the sleep-wake regulating process. Such influence can be accounted for with our model (Putilov, 1995).

The parameters of this model (Table 1, left column) were initially derived from the experimentally obtained durations of recovery sleep after six gradually increasing intervals of extended wakefulness (Åkerstedt and Gillberg, 1981), from data on the levels of SWA obtained for 10 naps (Dijk et al., 1987), and from data on two recovery sleep episodes (Dijk et al., 1990, 1991; see Putilov, 1995, for more details). X was expressed in relative SWA units (Putilov, 1995, 2014).

The model predicts that the level of SWA is gradually increasing during wake phase of the sleep-wake cycle. This increase is simulated as a reverse exponential function. However, due to the sine form (circadian) modulation of each of its parameters, the buildup of SWA occurs more or less rapidly. When the sleep homeostatic mechanism recognizes the deviation of the SWA level from its set point, the opposite – sleep – phase is initiated. This phase is simulated as an exponential function. Again, due to the sine form (circadian) modulation of each of the parameters of this function, the decay of SWA occurs more or less rapidly, and, again, the switching between two opposing phases occurs when the homeostatic mechanism recognizes the deviation of the SWA level from its set point (see Putilov, 1995, 2014, for more details).

The present simulations were performed by utilizing slightly modified parameters of this sleep-wake regulating process as compared to those derived from empirical data in the initial publication (Putilov, 1995). The plausible reasons for such modifications were the following. It is well-known that the amount of SWA dramatically reduces from childhood to old adulthood (Gaudreau et al., 2001; Gradisar et al., 2011; Campbell et al., 2012), that duration of sleep is also reduces in the same direction (Ohayon et al., 2004; Moraes et al., 2014), and that sleep timing is gradually delaying across adolescence until reaching the peak of lateness at age 18–20 years (Roenneberg et al., 2004; Fischer et al., 2017). Therefore, we took into account the fact of derivation of the parameters of the rhythmostat (Putilov, 1995) by using data on duration of sleep following extension of wakefulness from the experiment with six subjects in the age range of 29–45 years (Åkerstedt and Gillberg, 1981). Given younger age of participants included in 160 samples and given significant negative relationships of age with both SWA level and sleep times, we suggested the necessity to allow a higher SWA level in simulations for these ages (Table 1, right columns). This let us account for such features of the teen’s sleep as higher SWA levels, delayed sleep times, and longer sleep duration. Despite this, only a smaller fraction of parameters of the homeostatic process (1) was allowed to vary across most of ages (Tables 1, 2), and we did not propose any age-associated changes in the circadian term (2).

Because times in bed on weekends in the analyzed samples (Table 2) were similar to the experimentally determined maximal sleep capacity (8.9 ± 0.4 h) in participants of the same (18+) age in Klerman and Dijk (2008) study, we utilized data on weekend bed- and risetimes (*t1* and *t2*) and on weekday risetime (*t2* from Monday to Friday) as an input to the model. For the sake of simplicity and clarity, we rounded off all sleep times and most of other model’s parameters (Table 2).

## Results

In our simulations (Figures 1–3), bedtimes and risetimes were predicted as the switches between buildup and decay (wake and sleep) phases of the sleep-wake regulatory process that was disturbed on weekdays by social clocks. They required an earlier risetime than that expected in the case of socially unrestricted sleep. We postulated that bedtime at any of the days was determined exclusively by the sleep-wake regulatory mechanism, and that the same was true for the risetime on Saturday and Sunday mornings (Figure 3). In contrast, the risetime on Monday–Friday occurred earlier than expected in the case of socially unrestricted sleep, and this time was simply taken from the empirical dataset (Tables 1, 2). Table 3 contains the estimates of discrepancies between empirical and simulated sleep times. In general, they indicated that, by utilizing bed- and risetimes on weekends and weekday risetime as an input, rather accurate predictions of other sleep times (i.e., bedtime and time in bed on weekdays) were obtained. In particular, Figures 1, 2 illustrate that the discrepancies between the empirical and simulated sleep times were much smaller than the range of ±95% confidence interval for the empirical sleep times (Figure 2).

The simulations revealed contribution of the society (social clocks) to the pattern of age-associated changes in susceptibility to social jet lag. The maximum was reached at age 16+ (Figure 2), i.e., earlier than the peak of lateness was reached (Figures 1, 2). Thus, the development of sleep times in adolescents controlled by the homeostatic process is not fully identical to the development of their social jet lag determined by both the homeostatic process and social clocks. People of late adolescent age (16+) rather than young adults (18+) become most susceptible to social jet lag (Figure 2). Young adults suffered less mostly due to the abrupt delay of their weekday risetime on 1 h during the transition from age 16+ to age 18+. The simulations showed that the decrease of severity of social jet lag at this age might be less pronounced without such 1-h delay. It seems that these two age groups would have the severest social jet lag in the condition of identical weekday risetime. Although sleep timing further delays at age 18+, this delay is partly compensated by further sleep shortening (Figures 2, 3 and Table 3). Thus an increase in severity of social jet lag at this age relative to that at age of 16+ is prevented by the shorter sleep duration combined with later weekday risetime.

As we expected, the discrepancies for simulations of sleep times in eight age groups were found to be of a small size (Table 3) in those cases when the discrepancies between the results based on survey and actigraphy (Wolfson et al., 2003) were also small (Table 3). The best example of such small discrepancy is the weekend bedtime (Figure 2 and Table 3). In other words, small discrepancies between simulations and empirical data for the weekend bedtime were in agreement with small differences between actigraphic and survey sleep times (Table 3). In contrast, when the estimates for a sleep time obtained by Wolfson et al. (2003) with the methods of survey and actigraphy were very dissimilar (e.g., the difference was significant), the discrepancies between simulated and observed weekend bedtimes were large. This was the case for such a sleep time as the weekend risetime (Table 3). It was characterized by the largest difference provided by the comparison of survey and actigraphic data and the largest discrepancy in the comparison of simulated and empirical datasets. Importantly, these discrepancies between simulated and empirical datasets and this difference between survey and actigraphy were the same directional. Therefore, we concluded that the simulations confirmed the expectation that the weekend risetime might be overestimated in questionnaire datasets.

In simulation of sleep time in adolescents, it was not necessary to suggest any changes in SWA levels from the age 12+ to the age 18+ (Table 2). The differences between these ages were accounted for by the change in time constant of the wake phase (Figure 3B). Only the simulations of sleep times in younger ages required the correction of SWA levels toward their enlargement (Figures 1A,B and Table 2). In general, the simulations suggested that the time constant of the wake phase is increasing from childhood to adulthood. This increase became even more pronounced when the decline of SWA between ages 12+ and 18+ was incorporated in the simulations (this decline is likely to occur within this age interval).

While the time constant for the buildup (wake) phase showed the expected gradual increase across these adolescent’s ages, we did not reveal any systematic age-related change in the time constant for the decay (sleep) phase of the sleep-wake regulating process (Table 2). Thus, the buildup time constant was the only parameter of the model which modification was inevitable to simulate the delay of sleep phase during transition from the early school ages to the university students’ ages (Figure 3B and Table 2).

Notably, the identical age-related trend in sleep timing was observed in both M- and E-types. Despite approximately 2-h difference between chronotypes in sleep times in each of age groups, the general age trend for each chronotype closely resembled the trend showed by the whole set of data (Figure 1A). In fact, those who reported evening preference in younger ages were similar to those reporting morning preference in older ages on the age interval from childhood to late adolescence (Figure 1A).

In general, the simulations allowed the conclusion that the assumption of gradual change in just one of the model’s parameters, the time constant of wake phase of the sleep-wake regulatory process, might be sufficient for the explaining such phenomena as the prolongation of time staying awake and the reduction of sleep duration occurring in the course of transition from childhood to early adulthood.

We additionally noted that our simulation pointed at impossibility of sleep debt accumulation in terms of a further increase of SWA levels beyond the levels determined by the rhythmostat model (Figure 3C). Since such an accumulation does occur on weekdays, there is nothing to be repaid during weekends and vacation. Sleep becomes longer on Friday–Saturday night simply due to an earlier bedtime and it becomes longer on Saturday–Sunday night simply due to its initiation and termination at the same times as were expected in the case of spontaneous (socially undisturbed) sleep (Figure 3C). Therefore, the simulations suggested that there is no real way to recoup sleep lost caused the scheduling wakeups on weekdays on the early morning hours (Figure 3).

## Discussion

In this report we suggested a modeling framework for description age-related changes in sleep timing and duration. The most prominent of these changes is the dramatic sleep phase delay in the course of adolescence that was proposed to be linked to the increase in time constant of the buildup phase of the sleep-wake regulating process. The changes also include the related to this delay increase in severity of social jet lag in late adolescents as well as a decline of the level of SWA and decrease of sleep duration from childhood to adulthood. We simulated sleep times reported in the literature for 160 samples representing eight age groups to test the prediction that the ontogenesis of social jet lag can be accurately traced by using as an input to the sleep-wake regulating model the self-reported sleep times (i.e., weekday risetime and bedtimes and risetimes on weekends). The simulations accurately predicted other sleep times, i.e., the averaged bedtime and time in bed on weekdays, and allowed the determination of the particular parameters of the model underlying the maturational changes in homeostatic sleep regulation. The simulation results confirmed the hypothesis explaining the delay of sleep times by a shift in time constant of the reverse-exponential phase of accumulation of sleep pressure across wake episode. Thus, the results supported the expectation that adolescent’s sleep phase delay may not originate from alterations of the circadian timing system during puberty. Instead, the age-associated change in just one particular parameter of homeostatic rather than circadian component of sleep-wake regulation might be the major cause of the delaying shift of sleep timing during the transition from early adolescence to young adulthood.

Crowley et al. (2014) proposed that the differences in accumulation of homeostatic sleep pressure may allow the older adolescents to stay awake for a longer period of time thus delaying their bedtime. The simulations suggested that, indeed, the gradual change in just one of two time constants in the course of transition from childhood to early adulthood leads to the ontogenetic change in sleep-wake pattern that is characterized by the prolongation of time staying awake, the delay of bedtime, and the shortening of sleep duration and that is resulted in numerous troubles and tribulations for the maturating organisms.

Additionally, the model-based simulations provided the answers to the questions of how and when adolescents’ sleep times develop to become most susceptible to social jet lag (e.g., “Why adolescents are more susceptible to social jet lag than either young adults or children?”). Lateness reaches its peak somewhat later than does severity of social jet lag. However, the trajectory of development of sleep times due to the slowing down the buildup time constant of the homeostatic regulator indicated that adolescent’s susceptibility to social jet lag might be the highest due to earlier weekday risetime in comparison with the next age group (this time is 1 h earlier for 16+ than for 18+). It is expected that, if the social demands would continue to force young adults (18–23 years old) to wake up as early as they used to do in their school age (15–17 years old), they were suffering much more from social jet lag.

Consequently, it can be resumed that the growing organism is at greater risk of social jet lag due to three conditions that are typically combined at this age: (1) sleep times became later than in earlier ages, (2) sleep duration remains longer than in later ages, and (3) weekday risetime occurs as early as in earlier ages. Further development in the same direction in the early adulthood does not lead to a further increase in severity of social jet lag mostly due to the counterbalancing effect of the 1-h delayed shift of weekday risetime and further shortening of sleep duration.

We showed that the same values of parameters of the model (e.g., SWA levels) are suitable for predicting sleep times in most of separate age groups (e.g., between ages 10+ and 18+). However, the accuracy of prediction for some of the groups was not as good as for other groups. We did not try to further modify the parameters of the model to obtain a finer prediction because the accuracy of prediction was worse for only some of the predicted sleep time and it was much better for most other. We noted that a prediction remained always accurate for those sleep times that were not affected by the method of data acquisition in Wolfson et al. (2003) study. The example is the weekend bedtime. In contrast, a prediction was the least accurate for those sleep times which estimates were found to be significantly different in data provided by the methods of survey and actigraphy. The example is the weekend risetime that seemed to be overestimated in data obtained in survey of those adolescents who were characterized by late bedtimes. Therefore, we proposed that the imperfect (questionnaire) method of data collection might be an important contributor to the discrepancies between simulated and empirical sleep times obtained for these adolescents.

Another explanation for the highest discrepancies on weekend risetime obtained for age groups from 14+ to 16+ might be a phase delay of the circadian phase on the weekends. It is likely to be caused by the exposure to evening light, but it might be reversed by the counterbalancing exposure to morning light after 5 days of early morning awakening on weekdays. In this case, the delay of weekend risetime relative to the expected value might serve as a maker of this weekend delay of the circadian phase. In other words, the discrepancies between empirical and simulated sleep times for these age groups might be additionally explained by the delaying shift of the phase of circadian pacemaker that was not accounted for in the present simulations. The fact that such a delay most likely to occur in ages from 14+ to 16+ might be explained by their very early risetime on weekdays alternating with a relatively late sleep phase on weekends. The discrepancy for the weekend risetime for age 18+ might become smaller because such a shift of circadian phase caused by late sleep phase on weekends, if it really occurs, might become permanent due to a 1-h shift of the weekday risetime reported for this age group.

Notably, if the weekend shift due to evening light exposure followed by the opposite weekday shift due exposure to morning light really occur each week, it can be viewed as a typical jet lag rather than a social jet lag because both circadian pacemaker and sleep-wake timing are shifting back and forth. Moreover, as can be seen in Figure 1, there is, at least, one time point during the 24-h period when a phase of the sleep-wake cycle is predicted to remain identical for any day of the week. Namely, right before the time of socially imposed early morning awakening on weekdays the SWA level seems to be the same from Monday to Sunday (Figure 1). This modeling result questions the definition of social jet lag as the weekday-weekend shift of the sleep-wake cycle relative to the circadian pacemaker entrained by the external light–dark cycle during the week.

Finally, the simulations debunk the myth of catch-up sleep on weekends or vacation. They provided a clear answer to the question of “what is wrong with the idea of weekend catch-up sleep?” This idea presupposes weekday sleep is like a bank account, and that, if you run short for a while during weekdays, you need to get back what you lost when your schedule permits, e.g., during the following weekend or vacation nights. However, that is not the way the rhythmostat work. It does not serve for the accumulation of sleep debt during weekdays (e.g., in terms of further buildup of SWA level). Instead, it sends people to bed earlier than on weekends. If sleep has been lost by this particular way, it would not be caught up on the weekend. Getting extra sleep on two weekend nights to overcome the consequences of sleep restriction during five workday nights may seem like the right thing to do, but it is forbidden. What people think is their weekend extra sleep is exactly what would be expected to be just a normal sleep. In other words, the rhythmostat does not allow people to sleep longer than normal, and people’s weekend sleep remains of normal duration (determined by this internal device). On average, people are irretrievably losing slightly more than 1 h of sleep each weekday night (Figure 2) that is approximately two full weeks of slumber every year. On the other hand, being enforced to wake up earlier on weekdays, people are, in turn, awarded by, on averaged, two additional weeks of active life.

To resume, we proposed a methodological framework for the analysis of change in social jet lag and sleep timing during ontogeny. The studying simple mathematical models of the sleep-wake regulation allows making predictions about how adolescent susceptibility to social jet lag and late bedtimes is likely to develop during the transition from early adolescence to young adulthood. It tells us how age-related parameters of the sleep-wake regulating processes, such as levels of SWA, time constants, and sleep timing affect the trade-off adolescence’s organism may face between duration and timing of sleep on weekdays and weekends. The simulations predicted that adolescents’ susceptibility to social jet lag might generally be highest when their maturating organisms have sleep duration that is neither too short, nor too long in addition to sleep phase that is already late, but not the latest. The simulations also indicated that, throughout the ontogenesis, only one of the parameters of the homeostatic sleep regulator, namely, the time constant of the buildup (wake) phase, is required to be changed (to slow down) for explaining the cause of age-associated variation in the severity of conflict between their internal time keeping system and social clocks.

## Author Contributions

EV participated in discussion of the simulations and empirical data analysis, and made all computer simulations. AP proposed the idea and design of the simulation study, collected empirical data, made illustrations, and made the major contribution to the writing this paper.

## Conflict of Interest Statement

The authors declare that the research was conducted in the absence of any commercial or financial relationships that could be construed as a potential conflict of interest.
